# Influence of a Collapsed True Lumen in Acute Type A Aortic Dissection

**DOI:** 10.5761/atcs.oa.25-00148

**Published:** 2025-11-06

**Authors:** Kayo Sugiyama, Yuki Orimoto, Kazuma Kiryu, Hirotaka Watanuki, Masato Tochii, Akio Kodama, Hiroyuki Ishibashi, Katsuhiko Matsuyama

**Affiliations:** 1 Department of Cardiac Surgery, Aichi Medical University Hospital, Nagakute, Aichi, Japan; 2 Department of Vascular Surgery, Aichi Medical University Hospital, Nagakute, Aichi, Japan; 3 Department of Radiology, Aichi Medical University Hospital, Nagakute, Aichi, Japan

**Keywords:** acute type A aortic dissection, ascending aortic cannulation, total arch replacement with frozen elephant trunk, transesophageal echocardiography

## Abstract

**Purpose:**

We evaluated patients with acute type A aortic dissection (ATAAD) who were suffering from a collapsed true lumen in the descending aorta.

**Methods:**

Among 146 patients with ATAAD who underwent surgery, 6 (4.1%) with a collapsed true lumen of <10% of the total area at the level of the diaphragmatic transition in the descending aorta were detected. Preoperative and postoperative computed tomography images, preoperative characteristics, surgical techniques, and major adverse aortic events were assessed.

**Results:**

Patients with a collapsed true lumen tended to have spinal cord or peripheral malperfusion preoperatively. In two patients, because intraoperative transesophageal echocardiography showed no improvement in the collapsed true lumen after femoral artery cannulation, ascending aortic cannulation was added. Entry resection was achieved in five patients; however, three of them needed thoracic endovascular aortic repair (TEVAR). All six patients survived for one year, and after staged TEVAR, aortic remodeling was achieved.

**Conclusion:**

Patients with a collapsed true lumen in the descending aorta tended to develop lower body malperfusion, and usual cardiopulmonary bypass may be ineffective. Even if entry resection was achieved, aortic remodeling could not be obtained in some cases; therefore, staged repair with TEVAR can solve this issue.

## Introduction

Few reports have described the treatment of acute type A aortic dissection (ATAAD) with a collapsed true lumen in the descending aorta, and the prognosis of these patients remains unclear. Enlargement of the false lumen and compression of the true lumen can result in a critical condition.^1,2)^ Increased false lumen pressure may cause rupture of the descending aorta, while compression of the true lumen may impair systemic perfusion beyond the descending aorta, leading to lower body malperfusion.^3)^ However, coherent reports on appropriate surgical strategies for these patients are lacking.

Most cases of a collapsed true lumen in the descending aorta occur in type B aortic dissection, and extra-anatomic bypasses, such as axillary–femoral artery bypass or intravascular treatments including fenestration, have been effective. Recently, thoracic endovascular aortic repair (TEVAR) has gained popularity for managing complicated type B aortic dissections in patients with a collapsed true lumen.^4–8)^ However, few studies have addressed the treatment of ATAAD with a collapsed true lumen in the descending aorta,^1,9)^ and an appropriate strategy for cardiopulmonary bypass, including the cannulation site, has not yet been established. The primary goal in treating ATAAD is to save the patient’s life through central repair, including primary tear resection. However, whether central repair alone is sufficient to treat a collapsed true lumen of the descending aorta remains unclear.

In this retrospective study, we aimed to evaluate a case series of patients treated for ATAAD with a collapsed true lumen in the descending aorta.

## Materials and Methods

**Fig. 1:**
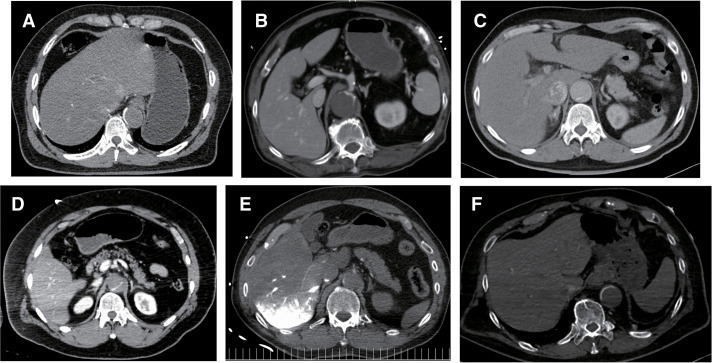
(**A**–**F**) Preoperative computed tomography images of each patient showing a collapsed true lumen.

Six patients with a collapsed true lumen comprising <10% of the total aortic area at the level of the diaphragmatic transition on preoperative computed tomography (CT, Revolution CT; GE Healthcare Inc., Chicago, IL, USA) were included in the study (**Figs. 1A**–**1F**). Many authors have discussed the ratio of the diameters between the collapsed true lumen and the distended false lumen^6,10–13)^; however, few have addressed the ratio of their areas.^14)^ According to Immer et al., although measured at 6 months postoperatively, a true lumen area of 30% or less has been shown to cause major aortic events, including long-term aortic dilatation.^14)^ While they used 30% as the cutoff value, we set a stricter threshold of 10% for measurements taken at the time of admission. Additionally, similar studies have discussed the ratio of true lumen to false lumen diameters which we also referenced.^6,10–13)^ Furthermore, Immer et al. analyzed the aortic diameter at the level of the pulmonary artery bifurcation, supra-diaphragm and infra-diaphragm, and the level of the infrarenal abdominal aorta, which we used as a reference.^14)^ Chen et al. also performed measurements at the diaphragm level,^10)^ although other literature defined it at the 8th,^13)^ 10th,^6)^ or 11th^12)^ intercostal space. These 6 patients accounted for 4.1% of the total 146 patients.

The extent of graft replacement was based on the location of the primary entry. For aortic arch replacement, the frozen elephant trunk (FET) technique was utilized. After the patient was anesthetized, intraoperative transesophageal echocardiography (TEE, EPIQ 7; PHILIPS, Amsterdam, the Netherlands) was used to evaluate cardiac function, aortic valve regurgitation, and cardiac effusion. TEE was also used intraoperatively to monitor the true lumen of the descending aorta at the level of the aortic valve. At our institute, femoral artery cannulation is standard for ATAAD surgery; however, additional cannulation of the ascending aorta or carotid artery was considered if there was malperfusion in the carotid arteries or if TEE showed inadequate flow improvement in the true lumen of the descending aorta at the level of the aortic valve. Selective cerebral perfusion was established during lower body circulatory arrest, with an intra-bladder or pharyngeal temperature target of 25°C. Continuous bilateral cerebral and extremity regional oxygen saturation was monitored throughout the surgery using INVOS Cerebral/Somatic Oximetry Adult Sensors (Medtronic, Minneapolis, MN, USA).

## Results

The preoperative characteristics of the 6 patients with a collapsed true lumen are shown in **Table 1**. None of the patients showed hemodynamic instability requiring intubation or inotropic support, and one patient showed moderate or severe aortic valve regurgitation. None of the patients had coronary or cerebral malperfusion, while half of the patients developed peripheral malperfusion, and one-third developed spinal cord malperfusion. Five patients presented an entry tear in the aortic arch, and the remaining one in the descending aorta.

**Table 1:** Characteristics of patients with a collapsed true lumen

Variable	1	2	3	4	5	6
Sex, male	Yes	Yes	Yes	Yes	Yes	Yes
Age (years)	50	69	49	55	65	78
Comorbidity						
Hypertension	Yes	Yes	Yes	Yes	Yes	Yes
Renal dysfunction	No	No	No	No	No	No
Chronic respiratory disease	No	No	No	No	No	No
Coronary artery disease	No	Yes	No	No	No	No
Cerebrovascular disease	No	No	No	No	No	No
Resuscitation	No	No	No	No	No	No
Cardiac tamponade	No	No	Yes	No	Yes	No
Aortic regurgitation III or IV	No	No	No	No	Yes	No
Malperfusion						
Cerebral	No	No	No	No	No	No
Coronary	No	No	No	No	No	No
Spinal cord	No	No	No	No	Yes	Yes
Mesenteric	No	No	No	No	No	No
Peripheral	Yes	No	No	Yes	Yes	No
Intimal tear in the descending aorta	No	No	No	Yes	No	No
Length of dissection in the descending aorta	Yes	Yes	Yes	Yes	Yes	Yes
Cannulation	FA, AxA	FA, AxA	FA	FA	FA, AscA	FA, AscA
Surgery	TARFET	HAR	TARFET	HAR	TARFET	TARFET
Entry tear resection, n (%)	Yes	Yes	Yes	No	Yes	Yes
Operation (min)	508	377	405	341	361	318
Cardiopulmonary bypass time (min)	269	209	215	165	234	159
Aortic cross-clamp time (min)	176	112	145	112	167	94
Hospitalization days	15	16	15	9	29	38
30-day mortality	No	No	No	No	No	No
Surgical morbidity						
Stroke, n (%)	No	No	No	No	No	No
Spinal cord injury (%)	No	No	No	No	No	Yes
Cardiac events, n (%)	No	No	No	No	No	No
Pulmonary failure, n (%)	No	No	No	No	No	No
Renal failure requiring dialysis, n (%)	No	No	No	No	No	No
Reentry remaining, n (%)	Yes (reentry in the abdominal aorta)	No	Yes (DANE)	Yes (entry in the LSCA)	No	No
All-cause mortality, n (%)	Alive	Alive	Alive	Alive	Alive	Dead (POD 295)
Major adverse aortic event (%)	TEVAR (POD 166)	None	TEVAR (POD 15)	TEVAR (POD 99)	None	None

AxA: axillary artery; AscA: ascending aorta; DANE: distal anastomotic new entry; FA: femoral artery; HAR: hemiarch replacement; LSCA: left subclavian artery; POD: postoperative day; TARFET: total arch replacement with the frozen elephant trunk technique; TEVAR: thoracic endovascular aortic repair

The operative procedures and outcomes are shown in **Table 1**. In two cases, additional ascending aortic cannulation was required alongside femoral artery cannulation to achieve sufficient perfusion because TEE showed a collapsed true lumen even after the establishment of cardiopulmonary bypass. Following this adjustment, TEE showed improvement in the impaired flow within the true lumen. Among the three patients with peripheral malperfusion, one exhibited lower body hypoxia as indicated by INVOS monitoring, which improved after additional ascending aortic cannulation. The remaining two patients presented normal INVOS data throughout the surgery. Entry resection was achieved in five patients; however, in one case, entry resection was not achieved because the primary entry was located in the descending aorta.

**Fig. 2:**
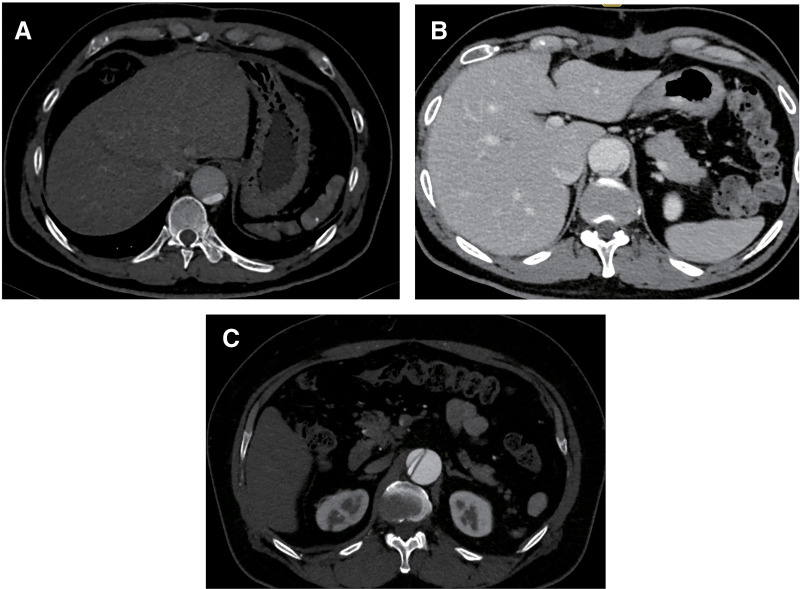
(**A**–**C**) Postoperative computed tomography images of 3 patients showing persistent collapsed true lumen.

**Fig. 3:**
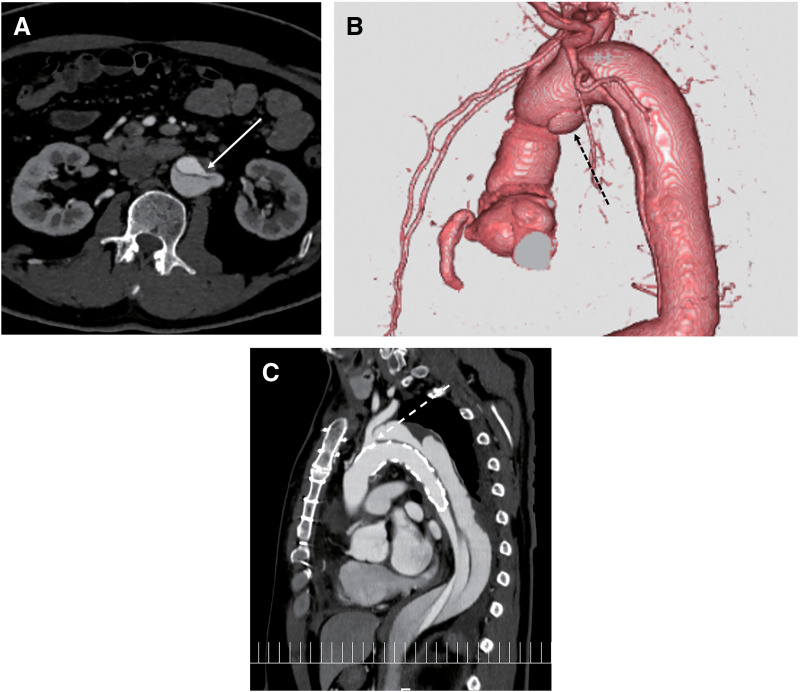
(**A**) Postoperative computed tomography image showing a persistent collapsed true lumen due to reentry in the abdominal aorta (white arrow). (**B**) Postoperative computed tomography image showing distal anastomotic new entry (black dotted arrow). (**C**) Postoperative computed tomography image showing a persistent collapsed true lumen due to an unresolved primary entry in the left subclavian artery (white dotted arrow) in a patient who underwent fenestrated aortic repair.

After surgery, spinal cord injury was observed in one patient who had presented with paraplegia before surgery, but peripheral malperfusion was improved in all three patients. There were no early mortalities, and all six patients were discharged without major critical complications related to the collapsed true lumen. During follow-up, one patient died from sepsis; however, no patients experienced aortic-related death. Postoperative CT imaging revealed a remaining collapsed true lumen in three patients (**Figs. 2A**–**2C**) due to the following causes: a small reentry in the abdominal aorta (**Fig. 3A**), a distal anastomotic new entry after hemiarch replacement (**Fig. 3B**), and an entry in the left subclavian artery after fenestrated total arch replacement (**Fig. 3C**). These three patients required TEVAR 3 months after aortic repair, resulting in good aortic remodeling.

All procedures were performed in accordance with the tenets of the Declaration of Helsinki. This study was approved by the Ethics Committee of Aichi Medical University Hospital on September 26, 2023 (approval number: 2023-482). Written consent was obtained from all patients for the use of their clinical data in conference presentations and publications.

## Discussion

Entry resection with central repair was effective for treating ATAAD with a collapsed true lumen. All patients survived and did not experience critical complications related to the collapsed true lumen. Some patients needed additional treatment because the collapsed true lumen was not resolved; however, aortic remodeling was achieved with additional TEVAR.

Patients with a collapsed true lumen tended to have lower body malperfusion involving peripheral and spinal cord arteries due to the collapsed true lumen in the descending aorta. Although these malperfusions can be resolved if the collapsed true lumen improves with central repair, it is sometimes difficult because of the irreversible ischemic damage. Spinal cord injury is a concern as bilateral intercostal arteries arise from the thoracoabdominal aorta to supply the spinal cord. When dorsal blood flow is disrupted due to a collapsed true lumen, the risk of spinal cord injury increases.^15,16)^ One patient in the present study developed permanent spinal cord injury, highlighting the difficulty in recovering spinal cord perfusion once infarction occurs. However, because the tolerance time for the lower limbs is rather long, permanent damage can be avoided using appropriate surgical techniques and intraoperative management. The optimal approach for managing spinal cord malperfusion in ATAAD remains unknown, although there have been a few successful cases that were treated with spinal fluid drainage before surgery.^17)^ Therefore, further investigation is warranted. Conversely, in the present case series, cardiac tamponade and coronary or cerebral malperfusion were not recognized; this indicates that the collapsed true lumen in the descending aorta is not related to complications occurring in the proximal aorta.

After establishing cardiopulmonary bypass, TEE evaluation of perfusion in the true lumen was crucial. If TEE shows no improvement in the collapsed true lumen, additional cannulation, such as in the ascending aorta, apex, or carotid artery, should be considered. Femoral artery cannulation is feasible in most ATAAD surgeries; however, in some cases, femoral cannulation alone may be insufficient to achieve adequate perfusion. Ascending aortic cannulation was effective in the present cases where the true lumen remains collapsed.

The optimal surgical strategy for a collapsed true lumen in the descending aorta remains controversial. Some authors recommend total arch replacement with the FET procedure (TARFET), as it promotes aortic remodeling.^10)^ However, TARFET can be more invasive, and complications related to the FET, including distal stent graft-induced new entry or spinal cord injury, should be considered.^18,19)^ The Ascyrus Medical Dissection Stent Hybrid Prosthesis (Artivion) has been designed to seal the distal anastomosis and expand the true lumen, thereby preventing the formation of a distal anastomotic new entry tear.^20)^ However, it is not suitable for patients lacking a distal landing zone and requires careful consideration in those with distal entry tears, where its use may not be optimal.^21–23)^ Based on these discussions, total aortic repair is not necessary during the initial surgery, and staged aortic repair concomitant with TEVAR is effective.

This study has several limitations. First, relatively few patients were included owing to the rarity of this condition. Second, this was a retrospective, single-center study that lacked randomization. A multi-institutional study is required to address these limitations. Third, patients with ATAAD who died before arriving at our hospital were excluded. Among these excluded patients, there were likely critically ill patients with collapsed true lumens. Finally, in CT scans obtained at admission or from other hospitals, some small entry tears could not be identified.

## Conclusion

Patients with ATAAD and a collapsed true lumen in the descending aorta tended to have lower body malperfusion, although they did not develop preoperative complications in the proximal aorta. Intraoperative TEE was effective for evaluating perfusion in the true lumen of the descending aorta, and additional ascending aortic cannulation enabled sufficient perfusion. In cases where aortic remodeling was not achieved with the initial surgery, staged repair with TEVAR proved effective for treating a collapsed true lumen.

## Declarations

### Data availability

Data sharing is not applicable to this article as no new data were created or analyzed in this study.
